# Design of a Foam-Actuated Nano-Emulgel for Perioceutic Drug Delivery: Formulation, Characterization, and Antimicrobial Efficacy

**DOI:** 10.3390/gels11050373

**Published:** 2025-05-20

**Authors:** Theresa P. K. Varughese, Poornima Ramburrun, Nnamdi I. Okafor, Sandy van Vuuren, Yahya E. Choonara

**Affiliations:** 1 Wits Advanced Drug Delivery Platform Research Unit, Department of Pharmacy and Pharmacology, School of Therapeutic Sciences, Faculty of Health Sciences, University of the Witwatersrand, 7 York Road, Parktown, Johannesburg 2193, South Africapoornima.ramburrun@wits.ac.za (P.R.); nnamdi.okafor@wits.ac.za (N.I.O.); 2 Department of Pharmacy and Pharmacology, School of Therapeutic Sciences, Faculty of Health Sciences, University of the Witwatersrand, 7 York Road, Parktown, Johannesburg 2193, South Africa; sandy.vanvuuren@wits.ac.za; 3 Wits Infectious Diseases and Oncology Research Institute, Faculty of Health Sciences, University of the Witwatersrand, 7 York Road, Parktown, Johannesburg 2193, South Africa

**Keywords:** nano**-**emulsion, nano**-**emulgel, azithromycin, periodontitis, foams, perioceutics, antimicrobial

## Abstract

Periodontitis is a prevalent oral condition worldwide. Azithromycin, a conventional lipophilic drug for periodontal treatment, often causes systemic side effects when administered orally. To address this, azithromycin-loaded nano-emulgels were developed using olive oil as a carrier within a xanthan gum aqueous gel phase. This oil-in-aqueous gel emulsion was actuated into a foam for localized drug delivery in gingival and periodontal disease. The solubility of azithromycin in various vehicles was tested, with olive oil showing the best solubility (0.347 mg/mL). Thermodynamic stability testing identified viable nano-formulations, with encapsulation efficiencies ranging from 98 to 100%. These formulations exhibited rapid drug release within 2–8 h. Muco-adhesion studies and ex vivo permeability tests on porcine buccal mucosa highlighted the beneficial properties of xanthan gum for local drug retention within the oral cavity. Antimicrobial efficiency was assessed using minimum inhibitory concentrations against various oral pathogens, where the formulation with equal surfactant and co-surfactant ratios showed the most potent antibacterial activity, ranging from 0.390 to 1.56 µg/mL. This was supported by the shear-thinning, muco-adhesive, and drug-retentive properties of the xanthan gel base. The study also examined the influence of the oil phase with xanthan gum gel on foam texture, rheology, and stability, demonstrating a promising prototype for periodontitis treatment.

## 1. Introduction

Periodontal disease is becoming increasingly prevalent in our world today, especially with a rise in high-sugar food products and limited access to oral health services within communities. Periodontal disease is prevalent in approximately 60% of the global population, with 24% suffering from severe periodontal disease [1,2,3]. This oral disease appears to be more common in individuals older than 65 years and lower- to middle-income populations, and as the years progress, so does the prevalence of periodontitis [3]. Periodontitis is a chronic inflammatory disease affecting the gums. It is associated with bleeding and inflamed gums, periodontal pocket inflammation, and in severe cases, tooth loss [4]. This oral condition is brought about by a lack of oral hygiene practices and regular brushing habits, smoking, obesity, certain medications, and, lastly, conditions such as diabetes [5]. The growth of biofilm-forming pathogens, such as *Streptococcus mutans*, within the oral environment leads to plaque formation on the surface of the teeth and initiation of host-mediated inflammatory responses. This build-up of bacteria within the periodontal pockets makes conventional treatment, such as scaling and root planing, insufficient due to its inaccessibility to reach areas deep within the periodontal pockets [6]. The inflammation leads to progressive erosion of the underlying bone structures, resulting in tooth loss. Thus, the treatment of periodontal disease within its preliminary stages is extremely pivotal in halting the condition’s progression into severe stages. Current treatment includes antibiotic and anti-inflammatory medications such as amoxicillin, metronidazole, and azithromycin, administered systemically via the oral route or loaded within periodontal chips and intrapocket devices—adjunct to scaling and root planing [7]. Orally administered therapies are associated with side effects, especially with antibiotics and anti-inflammatory drugs due to their exposure to the systemic circulation and limited accessibility within deep periodontal pockets and crevices, resulting in incomplete periodontal treatment [8]. Also, the larger concentrations of orally administered antimicrobials used for periodontal treatment may lead to antimicrobial resistance, compelling the use of stronger doses and harsher antimicrobials to provide effective eradication of pathogenic microbial loads and successful treatment outcomes.

Perioceutics involves the use of antimicrobial and anti-inflammatory therapy in addition to scaling and root planing to provide an improved periodontal prognosis. Perioceutics research currently explores localized periodontal treatment, focusing on periodontal pockets, with nanotechnology taking precedence due to its role in localized drug delivery within small tissue spaces. Nanotechnology encompasses a broad range of systems such as nanoparticles, nano-emulsions, nano-gels, and nanocomposites, amongst others, and allows for targeted drug delivery, increased bioavailability, and reduced side effects [9].

A nano-emulsion is a mixture of two immiscible liquids to form a homogenous and kinetically stable solution with the use of surfactants [10]. Nano-emulsions can either be oil-in-water (O/W) or water-in-oil (W/O), depending on which phase is dispersed into the other. Nano-emulsions are incorporated into drug delivery systems due to their ability to increase the solubility of lipophilic drugs and increase bioavailability, especially in the gastrointestinal (GIT) system, since they are easier to digest, increase permeability across membranes, and provide localized drug delivery [10,11,12]. The droplets are also more stable and less prone to negative effects that tend to plague emulsions, such as Ostwald ripening, creaming, sedimentation, and flocculation due to reduced gravitational pull between the droplets [13]. The nanosize improves the droplets’ kinetic stability and enables a higher loading capacity due to the presence of a larger hydrophobic domain available for binding [14].

Foams have exhibited better accessibility into crevices or cavities, better spreadability on surfaces such as the skin, increased bioavailability, and increased surface area despite low liquid volume [15]. Due to its airy and less viscous nature, the spreading of the foam alleviates any need for force when applying, thus reducing the discomfort associated with treatment application and increasing compliance.

Azithromycin, the drug of choice in this study, is a broad-spectrum macrolide derived from erythromycin and has an enhanced activity against Gram-negative bacteria. It has been used to treat periodontal disease through the administration of a 500 mg tablet once a day for three days, in addition to mechanical debridement and oral hygiene practices [16]. However, systemic intake of Azithromycin does lead to side effects such as QTc prolongation, slight hepatotoxicity, and GIT adverse effects such as nausea and diarrhea, amongst others [17].

Using the notion of perioceutics, a local drug delivery system for providing azithromycin delivery into the periodontal pockets was conceptualized. Such development of periodontal treatment at the targeted site reduces the risk of systemic side effects and requires lower concentrations of azithromycin to achieve therapeutic effects. Such systems also provide enhanced bioavailability of hydrophobic drugs, localized drug therapy onto the inflamed and sensitive gums and periodontal tissues, as well as protection of the drug itself from rapid enzymatic degradation [18].

Although nano-emulsions offer several benefits, their low viscosity leads to limited spreadability and inadequate retention on target tissues, which restricts their clinical utility [19]. Thus, the aim of this study was to combine the unique properties of nano-emulsions and foams into a foam-actuated nano-emulgel loaded with azithromycin that can deliver a drug directly into the periodontal pockets while adhering to the mucosal surface to increase retention time for antimicrobial activity. This study investigated suitable nano-emulsion components within a xanthan gum gel base to provide muco-adhesion and foam stabilizing properties.

## 2. Results and Discussion

### 2.1. Nano-Emulsion Formulation

Nine nano-emulsion formulations were developed using olive oil as the oil phase, Tween 80 as the primary surfactant, and Labrasol as the co-surfactant and foaming agent. Olive oil was selected as the oil phase based on its superior solubility for azithromycin compared to other oils tested (Figure S1, Supplementary Materials). Localized activity and targeted drug delivery to infected and inflamed gum tissues require prolonged resident time within the periodontal pockets; hence, muco-adhesive xanthan gum was incorporated to form a nano-emulgel. Foaming properties are favorable for drug delivery to hard-to-reach areas such as the periodontal pockets, as well as enhancing the ease of application within the oral cavity—this property was imparted by Labrasol. Mannitol is added as a foam stabilizer to modulate the foam stability index and dissipation. Lecithin was incorporated as a stabilizer to minimize Ostwald ripening and improve droplet stability [13].

All formulations were initially prepared under continuous stirring, followed by homogenization and sonication. Formulations F 1:9 and F 2:8 did not form clear emulsions after visual inspection and presented with turbidity and creaming. Upon the addition of azithromycin, xanthan gum, and mannitol, only F 5:5, F 6:4, F 7:3, F 8:2, and F 9:1 (featuring equal or greater quantities of Tween 80 to Labrasol) produced viable nano-emulgels with no turbidity and creaming upon visual inspection.

### 2.2. Encapsulation Efficiency

**Figure 1:**
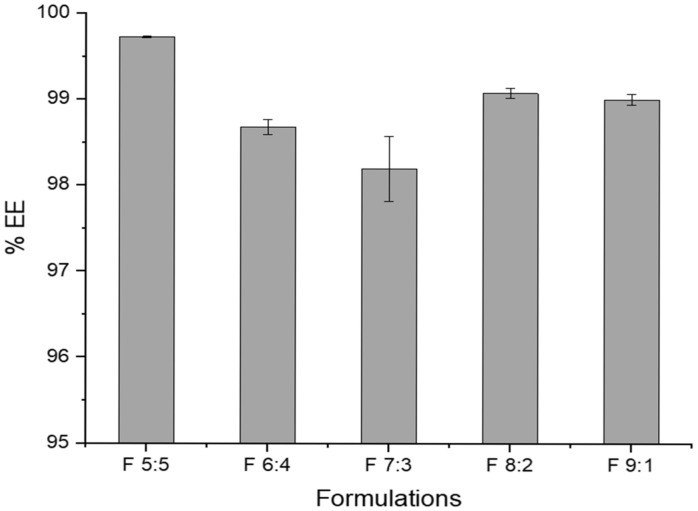
Quantification of the azithromycin encapsulation efficiency of nano-emulgels, where the ratios indicate the volumes of Tween 80 to Labrasol surfactant mix in each formulation.

The % encapsulation efficiency (%EE) of azithromycin in the nano-emulgels ranged from 98.2 to 99.7% (Figure 1). A high encapsulation efficiency is common in nano-emulgels due to various physicochemical properties of the drug and the components of the nano-emulgel. The oil droplets and the surfactants contain the lipophilic azithromycin molecules within the core of the oil droplets, whereas lecithin prevents drug leakage out of the core [20]. Lecithin is amphiphilic, having both hydrophilic and hydrophobic components, thus allowing azithromycin to be solubilized more readily within the dispersed phase of the nano-emulgel—enhancing the encapsulation efficiency [21]. Olive oil is rich in oleic acid (a lipophilic long-chain fatty acid), which facilitates the solubilization of azithromycin, thus resulting in a higher encapsulation efficiency whilst working synergistically with lecithin to increase drug encapsulation and stability of the nano-emulgels [22]. Labrasol, a lipophilic co-surfactant, further assists in solubilizing azithromycin within the olive oil carrier, leading to improved encapsulation.

F 7:3 depicts the lowest encapsulation efficiency amongst the formulations. This may be due to the presence of sufficient surfactant to form a micelle; however, not enough to stabilize the droplets and prevent the drug from partitioning out into the aqueous phase.

While having a high drug encapsulation is highly ideal, this may result in issues such as slight destabilization of the nano-emulgel due to high drug loading. To maintain reproducibility, strict control over process parameters such as mixing speed, temperature, and order of ingredient addition is required to ensure consistency.

### 2.3. Evaluation of Chemical Compatibility

FTIR analysis was conducted to identify interactions between the nano-emulgel components, excipients, azithromycin, and xanthan gum during nano-emulgel formation. Characteristic peaks of azithromycin, including O-H stretching at 3491 cm^−1^, C-H stretching of the alkyl group at 2974 cm^−1^, C-O-C ether stretching at 1189 cm^−1^, and the lactone ring at 1719 cm^−1^, were observed in the azithromycin-loaded nano-emulgels. As shown in Figures S2 and S3, Tween 80 and Labrasol exhibited similar FTIR spectra, indicating similar chemical profiles and properties, particularly in their polyethylene glycol chains and ester linkages. This supports their use as surfactant and co-surfactant, respectively, for forming the initial oil-in-water nano-emulsion [23].

The overlapping peaks of azithromycin with those of the nano-emulgel in the fingerprint region suggest that azithromycin is encapsulated and solubilized within the oil droplets of the nano-emulgel. Analysis of the nano-emulgel formulations (Figure S3G,H) revealed broad peaks from 3000 to 3700 cm^−1^, representing O-H stretching primarily due to the water content in the continuous phase. Tween 80, Labrasol, and xanthan gum also contributed to the O-H stretching signal in this region. Xanthan gum is evidenced by CH and CH_3_ stretching vibrations at 2900 cm^−1^, C-O stretching of carboxyl groups at 1600 cm^−1^, and C-O stretching of ether groups at 1100 cm^−1^. Tween 80 and Labrasol also contribute to the C-O stretching signal at 1100 cm^−1^ [24,25].

The FTIR analysis revealed no chemical interaction or chemical transitions (Figure S4) upon actuation of the nano-emulgel into a foam. The foam actuation process may have resulted in the rupturing of some nano-oil droplets within the emulgel formulation, exposing and dispersing azithromycin molecules within the bubble structure of the foam—thus exhibiting distinct peaks of azithromycin within the FTIR spectra of the foams as compared to that of the intact nano-emulgel, as listed in Table 1. These peaks are noted in the loaded foams at 2924 cm^−1^ and 1183 cm^−1^, respectively, depicting that the chemical integrity of azithromycin was maintained throughout the encapsulation and foam-actuation process.

### 2.4. Assessment of Physical Stability via Dynamic Light Scattering Properties

Dynamic light scattering was used to determine the formation and colloidal stability of nano-emulgel droplets. The nano-sized droplets enhance kinetic stability, preventing instabilities such as Ostwald ripening, flocculation, sedimentation, and creaming [26]. Surfactants reduce interfacial tension between oil and water phases, forming an energy barrier and creating an electric film between the dispersed droplets that stabilizes the system and reduces the rate of any destabilization [27,28]. In addition, the nanosize of the droplets allows them to overcome biological barriers, enabling targeted drug delivery [29].

The thermodynamic stability of the nano-formulations was assessed under three conditions: (1) centrifugation, (2) heat–cool cycles, and (3) freeze–thaw cycles. Table 2 and Table 3 compare the properties of the nano-emulsions and corresponding nano-emulgels (upon addition of xanthan gum base) for both drug-unloaded (blank) and loaded formulations before and after thermodynamic stability testing.

Formulations that changed appearance or exhibited creaming within 48 h were excluded. Eight out of ten blank formulations (containing no azithromycin) remained clear and stable after centrifugation, heat–cool, and freeze–thaw cycles (Table 2). Formulations F 3:7 and F 4:6 did not form clear nano-emulgels when loaded with azithromycin, likely due to oversaturation and precipitation from the hydrophobic core of the droplets. The drug may reduce the interfacial tension between the oil and aqueous phases by decreasing the available surfactant, thus destabilizing the nano-system [30]. The inclusion of xanthan gum and mannitol slightly reduced the stability index of azithromycin-loaded nano-emulgels, possibly due to disruption of the balance between components (oil, surfactants, and aqueous phase). The addition of xanthan gum gel brings the nano-emulsion closer to its water-titration endpoint, while the incorporation of azithromycin further disrupts the balance of the formulation components [30].

**Figure 2:**
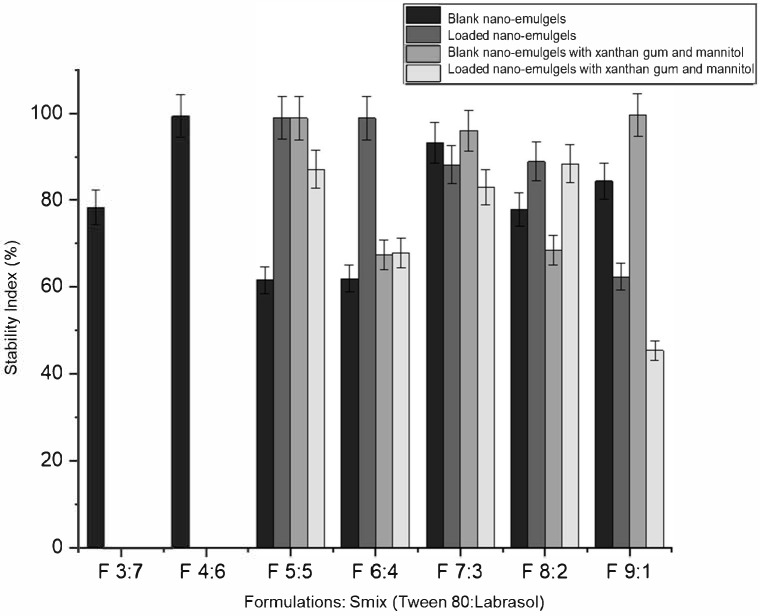
Stability index (%) of viable nano-emulgels after thermodynamic stability testing.

The remaining five nano-emulgels (F 5:5, F 6:4, F 7:3, F 8:2, and F 9:1) remained visually clear and stable with xanthan gum and mannitol, indicating no physical interactions with azithromycin. Blank nano-emulgels exhibited a slightly negative surface charge (as indicated by the zeta potential), which increased with azithromycin incorporation prior to and post thermodynamic stability testing. Xanthan gum, an anionic polymer, enhanced stability and improved polydispersity of the nano-formulations [31]. The addition of xanthan gum and mannitol improved thermodynamic stability, as shown in Table 3 and Figure 2. For example, F 5:5 B (blank) had a stability index of 61.59% and a zeta potential of 0.434 mV, while F 5:5 B+M+X (blank with mannitol and xanthan gum) had a stability index of 98.9% and a zeta potential of −8.65 mV. The zeta potential of the nano-emulgels trended towards more negative values after stability tests, except for the blank formulations. The addition of azithromycin influenced the zeta potential of the nano-emulgel formulations in a manner dependent on the surfactant mix (Smix) ratio. At Smix ratios of 5:5, 6:4, and 7:3, azithromycin loading led to an increase in zeta potential compared to the corresponding blank formulations, likely due to drug–surfactant interactions that enhanced surface charge stabilization. In contrast, at higher Smix ratios (8:2 and 9:1), a decrease in zeta potential was observed upon drug loading. This trend may be attributed to the higher concentration of Tween 80, a nonionic surfactant, which could reduce surface charge density or cause saturation at the droplet interface, thereby masking the charge contribution of the drug or other components such as anionic xanthan gum. These variations suggest that both the composition of the surfactant system and the drug’s localization at the interface play important roles in modulating the electrokinetic behavior of the nano-emulsion droplets.

A lower polydispersity index (PDI) indicates a more monodisperse and stable system. A monodisperse system tends to be more stable than a polydisperse system due to the uniform size of the droplets, thus preventing instabilities that may be caused by variations, such as temperature fluctuations [32].

As the concentration of Tween 80 increases, more positions on the hydrophobic hydrocarbon chains become available for azithromycin to interact with, reducing the critical micelle concentration needed for micelle formation and increasing thermodynamic stability [33]. Typically, micelle formation results in reduced droplet sizes due to surfactants assembling to solubilize azithromycin within their core, forming tighter, uniform micelles and resulting in a low polydispersity index (PDI), indicating monodisperse particle distribution [34].

Increasing the surfactant concentration also results in smaller droplet sizes, as observed with formulation F 9:1, where a higher Tween 80 concentration compared to Labrasol resulted in nano-droplets smaller than 12 nm. Micelle formation with non-ionic surfactants like Tween 80 and Labrasol often results in lower zeta potentials, as stability is imparted by steric hindrance rather than repulsion, unlike ionic surfactants [34]. Most nano-emulgels exhibited slightly negative zeta potentials (<−10 mV) before the addition of xanthan gum and mannitol. The addition of xanthan gum further increased the negative zeta potential, primarily due to the anionic nature of xanthan gum, forming an aqueous gel around the surface of each oil-containing micelle particle.

### 2.5. Effect of Drug Loading and Nano-Emulgel Composition on In Vitro Muco-Adhesion Properties

Mucin is a glycoprotein with a high percentage of carbohydrate chains (glycosylation), providing hydration and aiding in muco-adhesion. Xanthan gum, a natural hydrophilic polymer, interacts with mucin to enhance muco-adhesion through hydrogen bonding between their hydroxyl groups, forming a strong and stable interpenetrating polymer network at the mucosal layer where the free polymer chains diffuse into the mucus interface [31,35,36].

**Figure 3:**
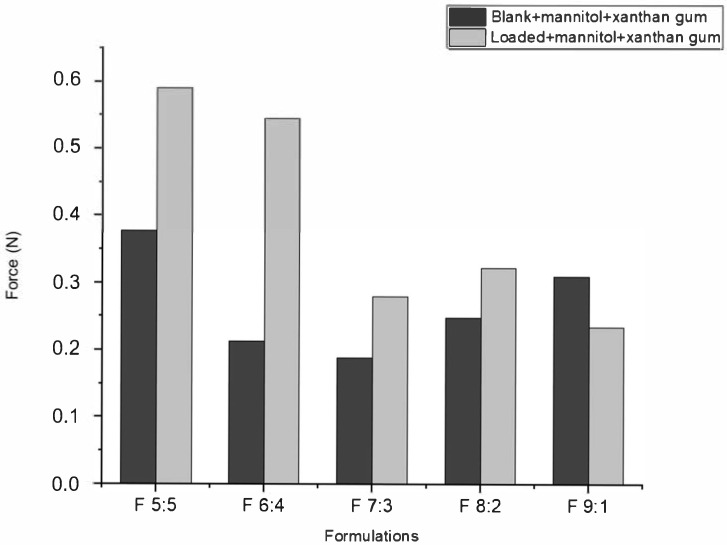
In vitro muco-adhesive behavior of nano-emulgels.

According to Figure 3, the muco-adhesion of nano-emulgels was influenced by azithromycin loading, the olive oil and surfactant mix ratio, and the stability index (Figure 2), typically displaying a muco-adhesive force of 0.1–5 N. Azithromycin-loaded nano-emulgels required greater force to detach from the in vitro mucosal model surface compared to their blank counterparts, except for F 9:1. This may be due to electrostatic interactions between the carboxylate group of mucin and the amine group of azithromycin, as well as interactions between azithromycin’s lactone ring and hydrophobic sites on the mucosal layer, enhancing retention and muco-adhesion [36]. Hydrogen bonds between the hydroxyl and ether groups of azithromycin and the glycoprotein network of mucin further strengthen muco-adhesive forces.

Olive oil, being hydrophobic, can interfere with the hydrophilic interactions between xanthan gum and the mucosal surface. While xanthan gum increases viscosity and stability in oil-in-water emulsions, a higher olive oil ratio may disrupt the hydrophilic gel network, reducing muco-adhesion [37]. The stability index correlates directly with muco-adhesive ability; a lower stability index, as seen with F 9:1, corresponds to lower muco-adhesion due to excess Tween 80 which may have interfered with polymer hydration and network formation in xanthan gum by competitively interacting with water molecules or via polymer–surfactant interactions (through hydrophobic interaction and hydrogen bonds), thereby reducing the ability of xanthan gum to interact effectively with mucin [38]. In contrast, F 6:4, with a lower surfactant ratio, displayed improved muco-adhesion and stability.

### 2.6. In Vitro Drug Release Studies

**Figure 4:**
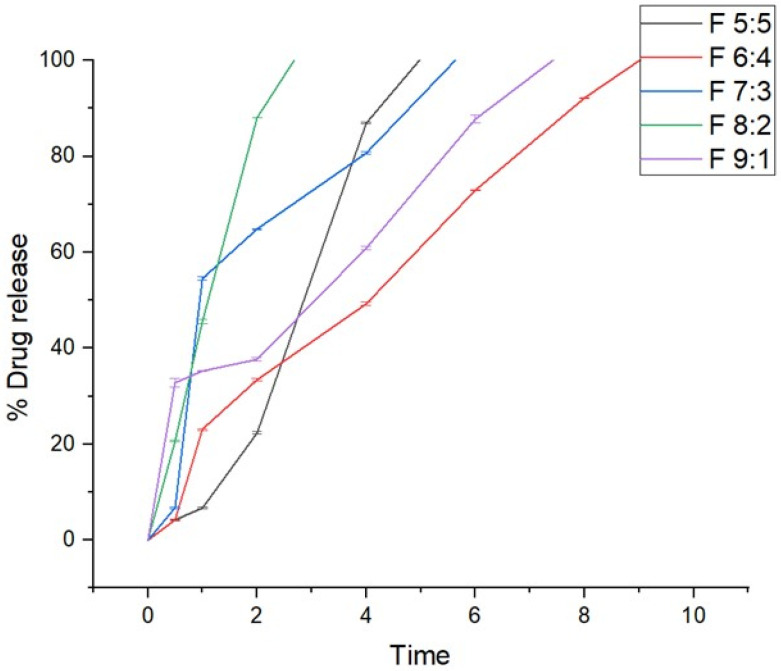
In vitro drug release profiles of azithromycin from the nano-emulgels (n = 3).

In vitro drug release studies were conducted to determine the release behavior of the nano-emulgels in simulated salivary fluid (SSF) of pH = 6.8, simulating the oral environment. These studies (Figure 4) showed that all formulations released within 2 to 9 h, with F 8:2 releasing rapidly within 2 h and F 6:4 taking the longest at 9 h. The rapid release observed in nano-emulgels is often due to the large surface area provided by the nano-droplet size. Higher zeta potential also influences drug release, with greater stability leading to faster and more consistent release.

The stability index (%) and release rate are directly proportional, where higher stability results in faster release. Nano-emulgel F 8:2, with the highest stability at 88.4%, exhibited the fastest release, likely due to high encapsulation efficiency and a larger stable surface area. Conversely, F 6:4 had the longest release time and the second lowest stability at 64.8%, displaying slight haziness indicative of phase separation. Phase separation can unevenly distribute the drug on the nanodroplet surface and within, leading to inconsistent and prolonged release [39].

### 2.7. Drug Release Kinetic Modeling

According to Table 4, most of the emulgels exhibit the Korsmeyer–Peppas kinetic model except formulations F 6:4 and F 9:1, which represent zero-order kinetics and first-order kinetics, respectively. The first-order kinetic model describes the drug release rate as dependent on the concentration of the drug available for release; higher concentrations result in faster release rates. Nano-emulgels with first-order kinetics allow for Fickian diffusion, where the gradual diffusion of the drug into the aqueous phase results in concentration-dependent release behavior. Zero-order release kinetics refers to a drug release mechanism in which the drug is released at a constant rate over time, regardless of its concentration. This means the same amount of drug is delivered per unit time, leading to a steady and predictable drug release profile. According to the Korsmeyer–Peppas model, n < 0.45 indicates transport through Fickian diffusion; 0.45 < 0.89 indicates non-Fickian transport; n = 0.89 corresponds with case II transport, whereas n > 0.89 corresponds with super case II transport. Since F 5:5 has an n-value of >0.89, it depicts a super case II transport, while F 7:3 depicts Fickian diffusion and F 8:2 non-Fickian diffusion [40].

### 2.8. Antimicrobial Studies

The oral microbiome is the second largest microbiome, primarily colonized by Gram-positive aerobes and facultative anaerobes such as *Streptococci*. Factors like poor oral hygiene, tobacco smoking, obesity, and hormonal changes can disrupt the normal oral microbiota, leading to dysbiosis characterized by an increase in Gram-negative anaerobes. This dysbiosis manifests as plaque accumulation, biofilm formation, bleeding gums, gingivitis, and eventually tooth and bone loss [41].

Oral pathogens such as *Streptococcus mutans*, *Lactobacillus casei*, *Lactobacillus acidophilus*, and *Aggregatibacter actinomycetemcomitans* significantly impact periodontal disease progression. Preventing or removing these infectious colonies is crucial to avoid systemic spread and severe diseases. *Lactobacilli* species contribute to oral disease when poor hygiene allows their colonization. *L. acidophilus* and *L. casei* produce lactic acid, lowering oral pH and promoting gingivitis-causing microbiota and dental caries [42,43]. The colonization of the oral cavity results in inflammatory conditions [44]. *A. actinomycetemcomitans*, a Gram-negative anaerobe, is known for causing periodontal disease, contributing to gum inflammation, bone resorption, and tooth loss [45].

The culture control consisted of the test pathogen grown in optimal media, confirming the viability of the organism through observed growth. The positive control was 0.1% azithromycin (showing antimicrobial susceptibility), and the negative control was 0.1% acetone (showing minimal inhibition).

Blank formulations exhibited antimicrobial activity comparable to or better than pure azithromycin (Table 5). The blank nano-emulgels (containing only oil, surfactants, and xanthan gum gel) exhibited antibacterial activity with an overall MIC of 1.56 μg/mL, except for 9:1, which exhibited an MIC of 0.78 μg/mL (Table 5). This activity may be attributed to the presence of olive oil and Tween 80. Olive oil has phenolic compounds that provide an antibacterial effect, whereas surfactants, such as Tween 80, may disrupt microbial cell membranes, causing lysis [22,46]. This inherent antimicrobial activity of the vehicle and surfactant may offer additional benefits in the clinical context, particularly for periodontal infections where biofilms and polymicrobial flora are involved. The synergistic effect between the vehicle and azithromycin could potentially enhance therapeutic efficacy, reduce required drug dosage, and minimize the risk of resistance.

Loaded nano-emulgels showed decreased antibacterial activity with increasing Tween 80 concentration, notably in *S. mutans* and *L. casei* for F 8:2 and F 9:1. This reduction is due to Tween 80 solubilizing hydrophobic antimicrobials into the aqueous phase, reducing their penetration into bacterial membranes. Additionally, Tween 80 promotes bacterial biofilm growth after primary adhesion [47].

Kinetic modeling indicated that F 5:5 follows the Korsmeyer–Peppas release mechanism, displaying super case II transport, allowing extended drug release at a constant concentration within the effective MIC range, resulting in the best MIC of 0.39 μg/mL. The remaining nano-emulgels exhibit first-order kinetic behavior, where the release rate depends on the available concentration. This behavior may influence MIC values differently, as declining concentrations may reduce the time available for complete bacterial eradication, causing drug concentrations to fall below the required MIC before inhibiting bacterial growth.

### 2.9. Rheological Behavior of Nano-Emulgels and Foams

Thixotropy is a time-dependent form of shear-thinning, where viscosity gradually decreases under constant shear stress and slowly recovers once the stress is removed. This delayed recovery is beneficial for topical and mucosal formulations, as it allows for ease of application followed by enhanced retention at the target site. Shear-thinning refers to the reduction in a material’s viscosity as shear stress increases, allowing the formulation to flow more easily under applied force, such as during application to a surface. Upon removal of shear, the material immediately returns to its original viscosity.

**Figure 5:**
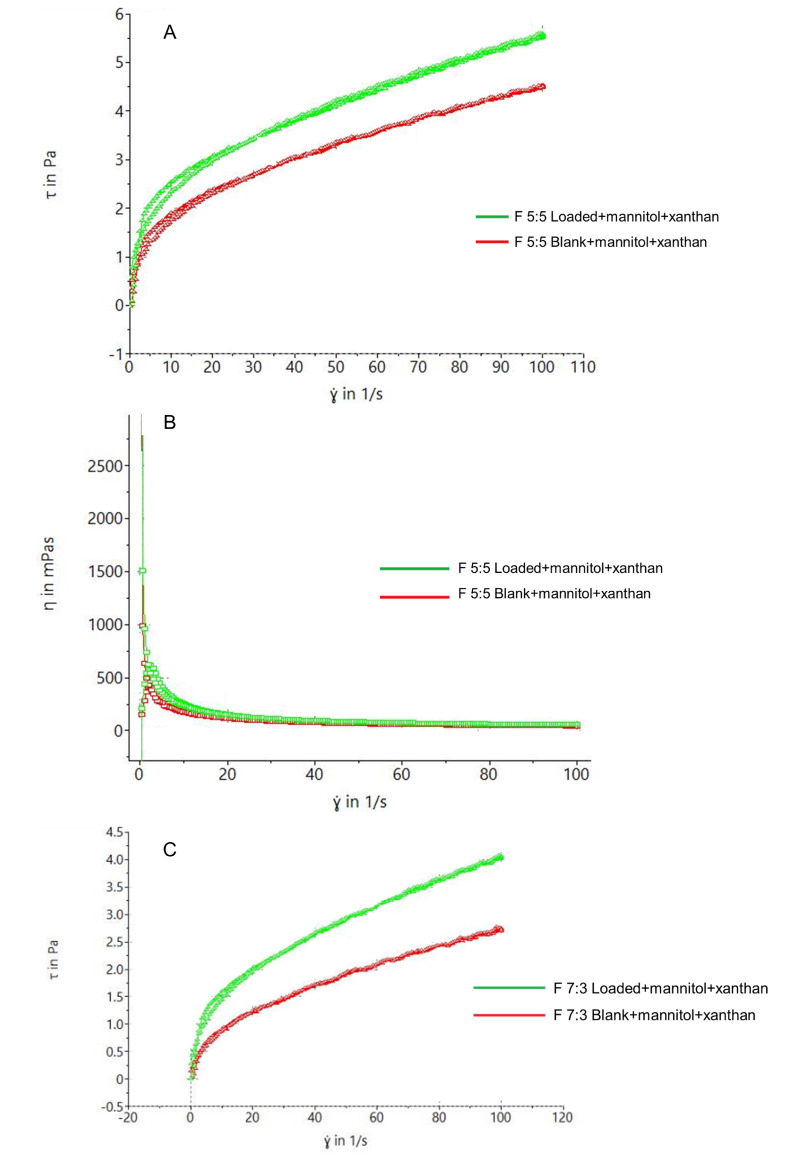

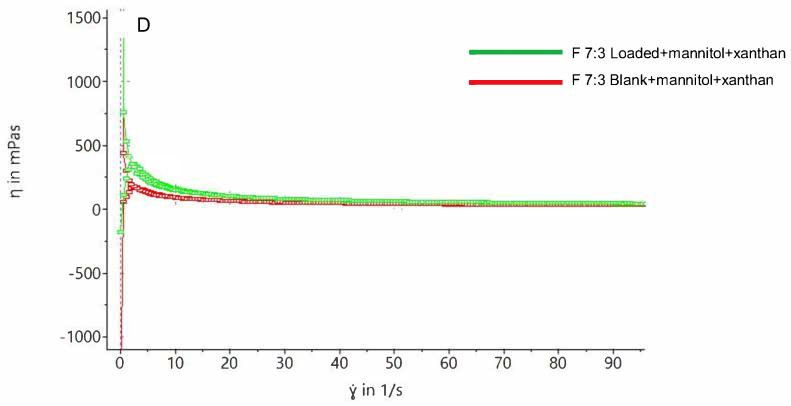
Rheograms of nano-emulsions showing (**A**) thixotropic behavior of F 5:5, (**B**) viscosity of F 5:5, (**C**) thixotropy of F 7:3, and (**D**) viscosity of F 7:3.

Thixotropy tests were conducted on the nano-emulgels and nano-emulgel-based foams to determine their shear-thinning behavior. Figure 5A,C show that F 5:5 exhibits slightly better thixotropic behavior, indicated by a larger hysteresis loop compared to F 7:3—this is crucial for formulation stability and retention at the application site. This is further supported by the viscosity vs. shear rate rheogram in Figure 5B,D, where F 5:5 starts with a viscosity > 2500 mPas, while F 7:3 starts at >1500 mPas, both displaying shear-thinning behavior with increasing shear rate. The foams exhibited significantly larger hysteresis loops due to their inherent shear-thinning behavior, enhanced by xanthan gum.

Naturally, the nano-emulgel depicted higher viscosity in comparison to the foams, due to the soft and airy nature of foams, thus resulting in lower viscosities in general.

**Figure 6:**
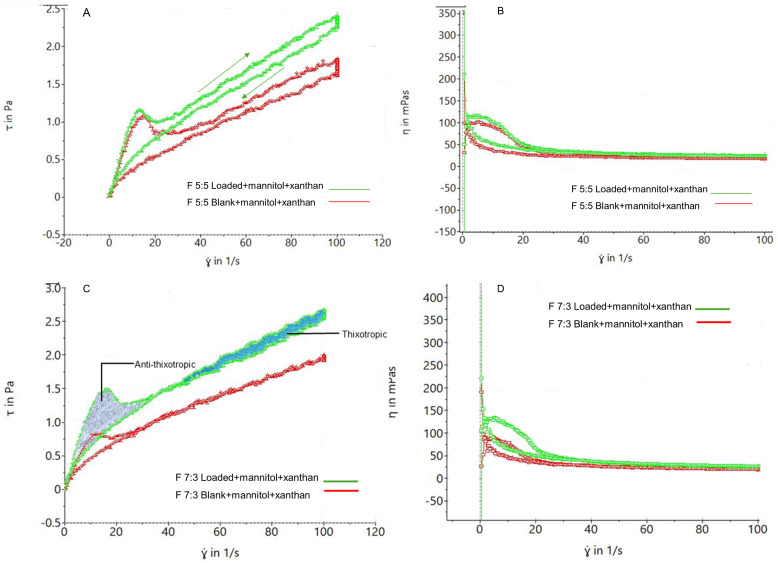
Rheograms of actuated foams showing (**A**) thixotropic behavior of F 5:5, (**B**) viscosity of F 5:5, (**C**) thixotropy of F 7:3, and (**D**) viscosity of F 7:3.

The nano-emulgel had higher viscosity compared to the foams due to the soft and airy nature of foams, resulting in generally lower viscosities. Figure 6A shows that both blank and loaded foams exhibit shear-thinning properties upon force application and revert to their original state upon force removal. This property is crucial for spreading the foam onto sensitive gums without applying excessive force. Increasing shear rate increases shear stress, while removing shear rate decreases shear stress. Figure 6B,D demonstrate that increasing shear rate decreases foam viscosity, reaching a minimum viscosity plateau, confirming the shear-thinning property.

Sample F 5:5 in Figure 6A illustrates the visco-elasticity of the foams, with shear stress increasing with shear rate and returning to the initial shear stress, forming a hysteresis loop. This loop indicates thixotropic behavior, with foam bubbles remaining relatively intact after stress application. Figure 6C shows the coexistence of thixotropic and anti-thixotropic behavior, as seen in F 7:3, where bubble deformation leads to anti-thixotropic behavior in less visco-elastic foams [48]. Due to its foam action, higher Labrasol concentration in F 5:5 may result in more visco-elastic foams compared to F 7:3, which has a lower Labrasol concentration.

### 2.10. Assessment of Foam Properties Actuated from Nano-Emulgels

**Figure 7:**
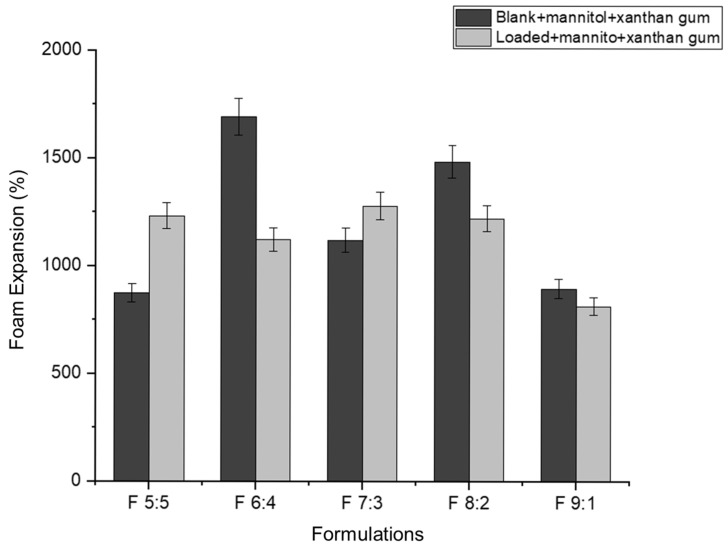
Foam expansion of viable nano-emulgel formulations.

**Figure 8:**
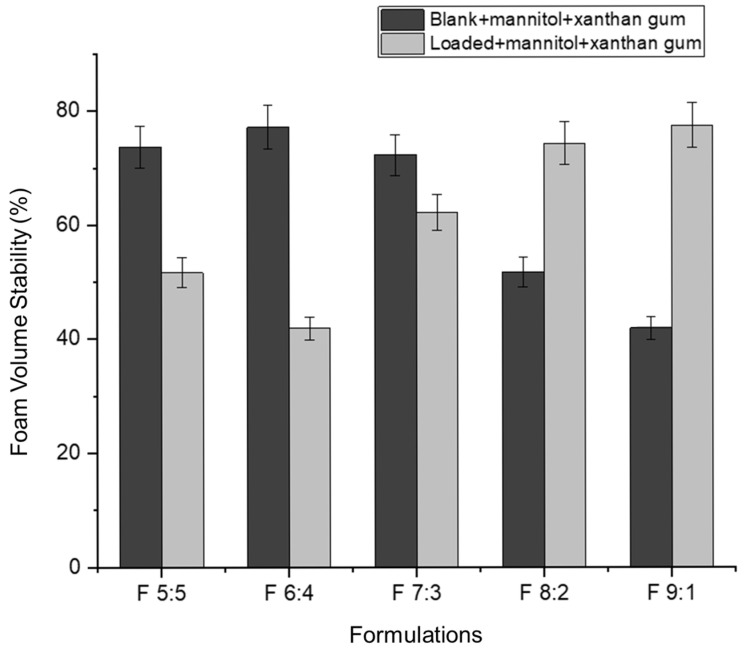
Foam volume stability of the actuated nano-emulgels.

Foam expansion refers to the volume of foam produced from a given volume of liquid [49]. In this study, approximately 5 mL of nano-emulgel (equivalent to five pumps with an actuator) was used to determine foam expansion. Foam expansion appears to have an inversely proportional relationship with foam volume stability (Figure 7 and Figure 8). As the percentage of foam expansion increases, foam volume stability decreases. This may be due to the formation of numerous but weaker bubbles, as most surfactants are used to form bubbles, leaving less surfactant available to stabilize them. Consequently, the thin films surrounding each bubble rupture easily under forces such as gravity, leading to rapid bubble collapse.

Based on Figure 8, foam volume stability improves in the loaded formulations with an increased concentration of Tween 80 relative to Labrasol. This can be attributed to the hydrophobicity of the hydrocarbon chains in Tween 80. According to Liang (2024), Tween 80 has a twist in its hydrocarbon chain due to a double bond, allowing better hydrophobic interactions between Tween 80′s hydrocarbon chains and azithromycin’s macrolide ring [33]. This leads to the formation of smaller nano-droplets, increasing the foam’s mechanical strength. The nano-droplets adsorb onto the excess surfactant in the bubble film, decreasing the drainage rate and preventing bubble rupture [50].

The inclusion of azithromycin in F 5:5 and F 6:4 may have resulted in larger droplets, exceeding the available surfactant for bubble film stabilization, thus causing quicker bubble rupture.

**Figure 9:**
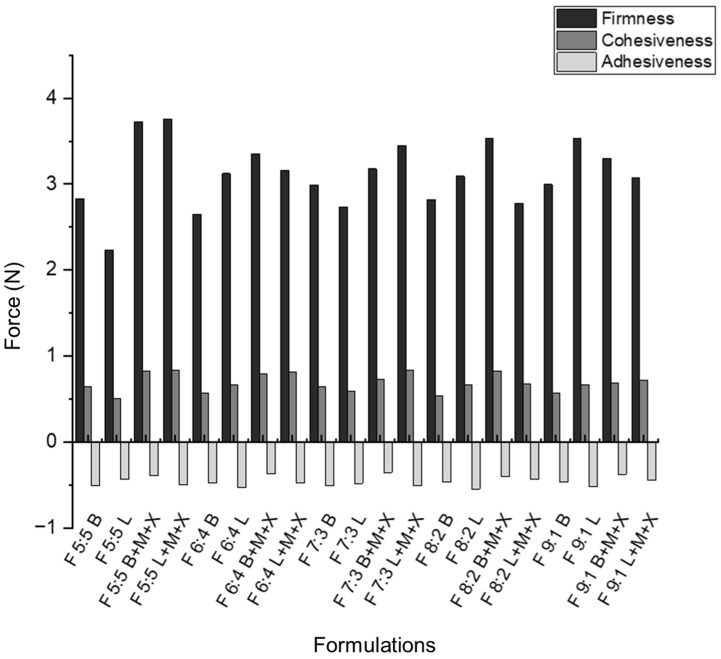
Firmness, cohesiveness, and adhesiveness of actuated nano-emulgel foams obtained from textural profiling.

The firmness, cohesiveness, and adhesiveness of the nano-emulgels were determined using the TA.XT plus texture analyzer (Stable Microsystems, Surrey, UK) [51], as shown in Figure 8 and Figure S4. Foam firmness was measured as the positive peak on the texture analysis graph [52]. Cohesive forces were calculated as the area under the curve (AUC) of the force–time graph. The inclusion of azithromycin in the nano-emulgels appears to strengthen the internal forces within the foams (Figure 9). This is supported by Figure S4, where the blank foam dissipates after the post-test run, while the loaded foams remain intact, indicating better foam strength and stronger cohesive forces.

The rheogram of F 5:5 supports the texture results, showing that this foam exhibits thixotropic behavior, returning to its original form with minimal deformation, indicating strong cohesive forces. Less foam expansion correlates with greater cohesiveness, as larger, non-uniform bubbles tend to have weaker structural integrity (Figure S5) [53].

Foam adhesiveness was determined by calculating the AUC for the negative peak of the texture analysis graph. Adhesiveness was tested between the instrument platform and a 100 mm flat probe, with results following the same trend as the muco-adhesive tests conducted on the nano-emulgels and foams. This indicates that foam actuation does not cause significant chemical or physical changes, except for slight rupturing of the nano-droplets, as evidenced in the FTIR spectra of the loaded foams (Figure 6). A balance between foam firmness and cohesion is essential for developing foams that are easy to spread within the oral cavity without exerting excessive force on sensitive gums, while maintaining enough structural integrity to prevent immediate dissipation after actuation.

### 2.11. Ex Vivo Muco-Adhesion of the Foaming System

Muco-adhesion allows drug delivery systems to attach to mucosal surfaces, typically through binding to mucin. In this foaming system, muco-adhesion enables nano-droplets to bind to the buccal mucosa, providing longer retention time and improving therapeutic potential by ensuring complete drug release at the target site. This suggests that foam actuation does not significantly alter the properties of the nano-emulgels, as their behavior remains similar before and after foam formation.

**Figure 10:**
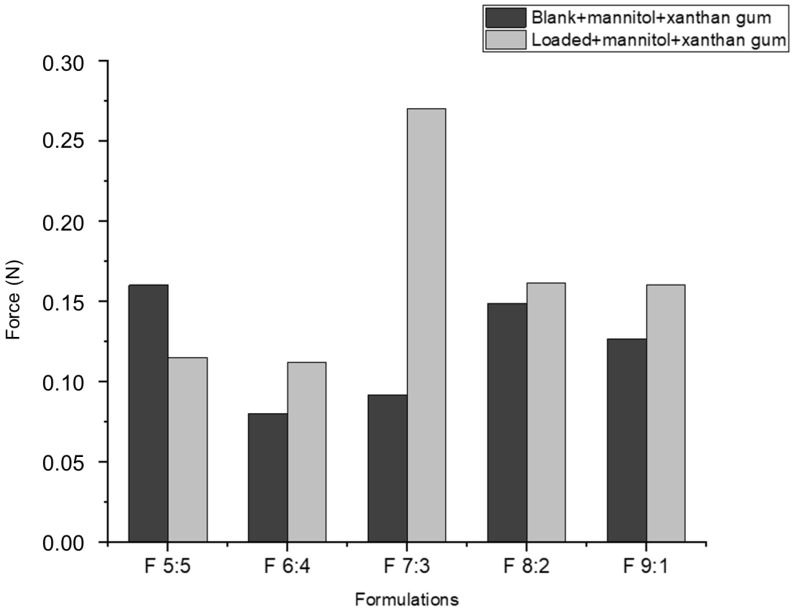
The ex vivo muco-adhesive behavior of foams quantified using the texture analyzer and porcine buccal mucosa.

Loaded foams with xanthan gum and mannitol (Figure 10) generally exhibit greater muco-adhesion compared to blank foams with the same components. This behavior is consistent with the muco-adhesiveness observed in nano-emulgels (Figure 3). Although both nano-emulgels and foams show better muco-adhesion in loaded formulations, the muco-adhesive force of nano-emulgels is significantly higher than that of foams. For instance, the F 6:4 loaded NE exerted a force of 0.054 N, while the F 6:4 foam exerted only 0.12 N. This difference may be due to the presence of air bubbles and liquid drainage in foams, which weaken the overall cohesive force compared to nano-emulgels. The nano-emulgels have stronger intermolecular and interfacial forces between the droplet and oil–water interface, due to the surfactants present [54].

### 2.12. Ex Vivo Permeability Studies

Porcine buccal mucosa was used to assess the permeability of the nano-emulgels, determining their ability to provide targeted and localized treatment by remaining within the tissue and preventing systemic drug release. This approach helps avoid systemic side effects and reduces the risk of antimicrobial resistance.

**Figure 11:**
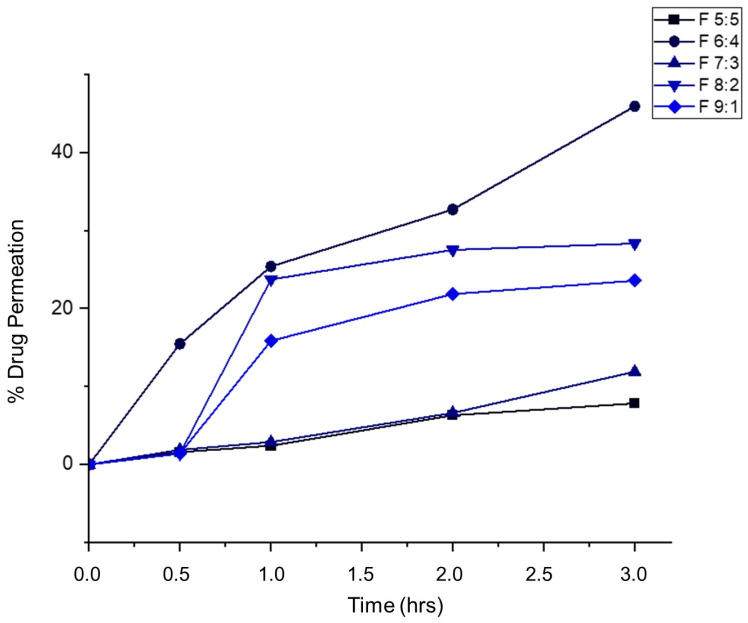
Ex vivo permeability of azithromycin from foams through porcine buccal mucosa.

As illustrated in Figure 11, less than 50% of the drug permeated through the buccal mucosa, with most of the drug retained within the tissue. This indicates that the drug delivery system provides a localized effect, as the drug is dispersed within the lipid bilayer of the buccal mucosa. Formulation F 5:5 showed the least permeation, with less than 15% of the drug penetrating through.

Muco-adhesiveness appears to correlate with ex vivo permeability; as muco-adhesiveness increases, drug permeation through the buccal mucosa decreases. Sample F 5:5, despite having a relatively low muco-adhesive force (0.21 N), had the least drug permeation (7.9%). The cohesiveness of the foams significantly influences permeability; stronger cohesion results in less drug permeation, likely due to the cohesive forces making it harder for the foams to break down and penetrate the buccal mucosa.

Formulation F 6:4 provided extended release for around 9 h and permeated the most into the buccal mucosa. This may be due to increased residence time, allowing more time for drug release into the periodontal pockets and penetration through the mucosa. The firmness and cohesive forces of F 6:4 are relatively weak compared to F 5:5 and F 7:3, facilitating quicker and deeper permeation. Additionally, higher concentrations of Tween 80, as seen in F 9:1 and F 8:2, can increase permeability, with these formulations permeating around 20–25% through the buccal mucosa and exhibiting weaker cohesive forces.

## 3. Conclusions

In this study, 10 nano-emulgels were formulated, with 5 proving viable after thermodynamic stability tests. Among these, F 5:5 emerged as the most optimal nano-emulgel, with a high stability index of 87.1% when loaded with xanthan gum and mannitol. It exhibited the highest encapsulation efficiency and a drug release rate of 4.5 h, ensuring adequate residence time within the gingival crevices. Approximately 8% of the drug permeated the buccal mucosa, ensuring a localized effect within the oral cavity. Both blank and loaded F 5:5 emulgels demonstrated strong antibacterial activity against the tested oral pathogens.

This study demonstrated that foams are a novel method for delivering antibiotics, antifungals, and other drugs. The foam aerated structure offers a large surface area and porous architecture that enhances contact with the gingival mucosa and periodontal crevices, promoting sustained drug release and improved local drug concentration. The emulgel-based foam formulated in this study aims to treat periodontal disease, offering a less painful treatment option for patients with gum sensitivity, bleeding gums, and inflammation. Its shear-thinning and muco-adhesive properties, supported by xanthan gum, aid in ease of spreadability and mucosal retention in an inflamed oral environment. Together, these features enhance the local bioavailability and antimicrobial efficacy of azithromycin against periodontal pathogens. The foam system has the potential to release azithromycin into gingival crevices and periodontal pockets through the formation of microbubbles and their dissipation into a muco-adhesive gel coating on target tissues.

Compared to conventional periodontal gels or foams, the proposed nano-emulgel foam formulation offers several advantages. Most commercial gels used for periodontitis, such as chlorhexidine gels, require frequent application and often suffer from limited access and muco-adhesion, particularly within periodontal crevices, and rapid clearance by salivary flow. Traditional foams, while easier to apply, generally lack sustained drug release. With a sustained release profile of up to 4.5 h, enhanced targeting, and muco-adhesion, this formulation ensures prolonged therapeutic action, reducing dosing frequency. The foam can be applied topically to the gingival margin using a soft applicator or clean finger, allowing precise delivery and better patient acceptability. Overall, this dual-function system presents a promising and patient-friendly option for periodontal therapies.

## 4. Materials and Methods

### 4.1. Materials

Labrasol ALF was procured from Carst & Walker (Johannesburg, South Africa). Olive oil, avocado oil, blackseed oil, and coconut oil were sourced from MN Globex (Indore, India). Azithromycin was purchased from DB Fine Chemicals (Johannesburg, South Africa). Porcine mucin Type II, cellulose dialysis tubing MWCO 12,000 Da, Tween 80, Tween 20, xanthan gum, mannitol, lecithin, PEG 400, and perchloric acid (70%) were obtained from Sigma-Aldrich (St. Louis, MO, USA). Mueller Hinton Broth, yeast extract**,** Haemophilus supplement, and Tryptone Soya Broth were from Thermo Fisher Scientific (Johannesburg, South Africa). The bacterial strains *Streptococcus mutans* (ATCC 25175)*, Aggregatibacter actinomycetemcomitans* (ATCC 33384)*, Lactobacillus acidophilus* (ATCC 4356), and *Lactobacillus casei* (ATCC 25598) were from the American Type Culture Collection (ATCC, Manassas, VA, USA).

### 4.2. Methods

#### 4.2.1. Preparation of Nano-Emulgels

**Figure 12:**
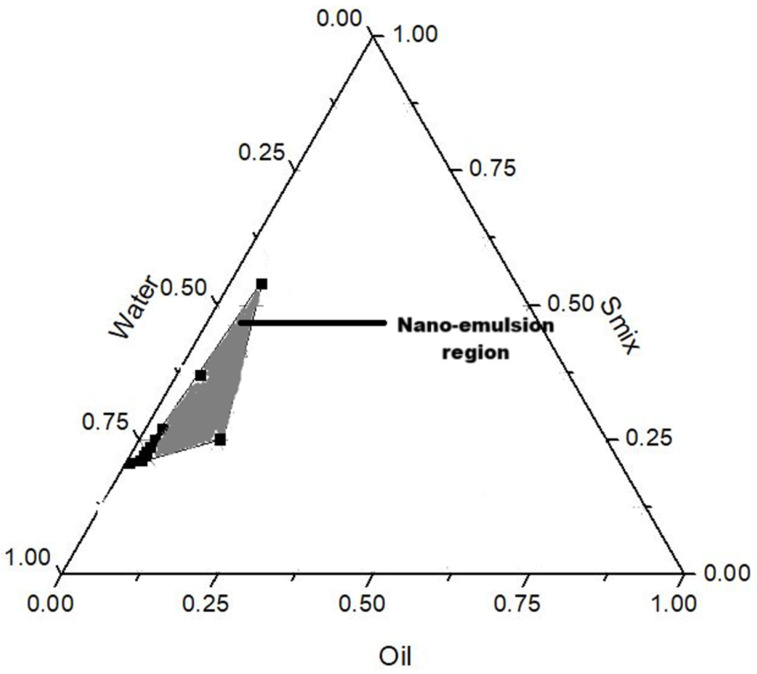
Pseudo-ternary diagram representing the viable nano-emulgel region according to volume fraction of oil, water, and surfactant mix (Smix).

Solubility studies in various oils and surfactants showed optimal azithromycin solubility in olive oil, Labrasol, and Tween 80 (S1). Oil-in-water nano-emulsions were prepared using a low-energy method where each component was mixed in various ratios with water as guided by the nano-emulsion forming region in the pseudo-ternary diagram (Figure 12), and as listed in Table 6. The oil, surfactant mix (Smix, referring to the volume ratio of Tween 80 and Labrasol), and lecithin were weighed into a beaker and heated under continuous stirring at 450 rpm until the lecithin had completely dissolved. This was followed by the addition of 0.1% *w*/*v* of azithromycin with further stirring until fully solubilized. Distilled water, as the aqueous phase, was added to the mixture and stirred until no phase separation was noted. Xanthan gum (1.5% *w*/*v*) was added to the resulting nano-emulsions and homogenized for complete solubilization to form nano-emulgels. Mannitol was added at a concentration of 0.5% *w*/*v*, and the system was sonicated for 30 min to enhance the stability of the formulation. The resulting nano-emulgels were placed into a foam actuator container and pumped to produce a foam (where one pump equated to 1 mL of nano-emulgel actuated into foam).

#### 4.2.2. Assessment of Chemical Compatibility

Chemical compatibility of the materials used in the nano-emulgel formulations was assessed using a Perkin Elmer Attenuated Total Reflectance-Fourier Transform Infrared Spectrometer (ATR-FTIR) (PerkinElmer 100, Llantrisant, Wales, UK). Samples were analyzed over a wavenumber range of 4000 to 650 cm^−1^, with spectra recorded as an average of 20 scans. A background scan was performed before each measurement to eliminate environmental noise.

#### 4.2.3. Assessment of Dynamic Light Scattering Properties

Dynamic light scattering (DLS) was performed using a Zetasizer Nano ZS (Malvern Instruments, Malvern, UK) to determine the nanodroplet size, zeta potential, and polydispersity index (PDI) of the nano-emulgel formulations. For analysis, 1 mL of the nano-emulgel was diluted with 10 mL of distilled water and filtered through a 0.22 μm PVDF syringe filter. The filtered sample was transferred into a disposable plastic cuvette. Measurements were conducted at 25 °C using a 4 mW laser at a fixed scattering angle of 90°, and the corresponding values were recorded.

#### 4.2.4. Thermodynamic Stability Testing

The thermodynamic stability of nano-emulgels was assessed through centrifugation, heat–cool, and freeze–thaw cycles. For centrifugation, 10 mL samples (n = 3) were placed in 15 mL tubes and centrifuged at 1000, 2500, and 3500 rpm for 30 min. Visual inspections for phase separation followed each speed. Samples passing centrifugation underwent six heat–cool cycles: 24 h at 37 °C in an orbital shaking incubator (LM-530-2, MRC Laboratory Instruments Ltd., Hahistadrut, Holon, Israel), followed by 24 h at 4 °C in a refrigerator. After six cycles (12 days), visual inspection was repeated. Stable samples then underwent six freeze–thaw cycles: 24 h at −20 °C followed by 24 h at 25 °C. After each cycle, samples were visually checked for instability. Formulations passing all cycles were reanalyzed by DLS to assess changes in size, PDI, and zeta potential. These parameters, along with physical appearance, were used to evaluate stability [55].

#### 4.2.5. Determination of Drug Encapsulation Efficiency

To determine the % encapsulation efficiency (%EE) of azithromycin, a nano-emulgel sample of 3 mL was centrifuged at 4000 rpm for 10 min and the collected supernatant was read using a UV–Visible spectrophotometer (Varian Cary UV–Visible Spectrophotometer 50 Conc, Agilent Technologies, Santa Clara, CA, USA) at 482 nm after oxidation with perchloric acid (n = 3). This change in the UV detection wavelength from 209 nm in the solubility studies is attributed to the nano-emulgel matrix reducing the absorption of azithromycin compounds at 209 nm, hence hindering detection [56]. Due to the lack of a strong chromophore in azithromycin, 70% *v/v* perchloric acid was used as an oxidizing agent to produce a detectable compound. The absorbance of the oxidized azithromycin was measured at 482 nm using a UV–Visible spectrophotometer (Varian Cary UV–Visible Spectrophotometer Con 50, Agilent Technologies, Santa Clara, CA, USA) [57,58]. The %EE was calculated using the following equation [59,60]:

$$\%EE=\frac{Total\;drug-Free\;drug}{Total\;drug}\times 100\%$$

#### 4.2.6. Assessment of In Vitro and Ex Vivo Muco-Adhesion

Muco-adhesive properties of the nano-emulgel and foam formulations were evaluated using both in vitro and ex vivo methods with a TA.XT Plus Texture Analyzer (Stable Micro Systems, Surrey, UK).

In the in vitro study, filter paper pre-wetted with a 0.5% *w*/*v* mucin solution was used as a mucosal substitute. The nano-emulgel was compressed using the texture analyzer in muco-adhesion mode.

For the ex vivo assessment, porcine buccal mucosa was used, obtained in accordance with Waiver 16-11-2023-O from the Animal Research Ethics Committee of the University of the Witwatersrand. To simulate the mucosal environment, a 0.5% *w*/*v* porcine mucin solution was applied to sections of the porcine buccal mucosa. The tissue sections were mounted onto a ½-inch cylindrical probe, and a sample of the nano-emulgel formulation was placed on the analyzer platform.

In both studies, the probe was brought into contact with the sample for 60 s at a test speed of 1 mm/s and a withdrawal post-test speed of 0.5 mm/s to determine the force of detachment (N).

#### 4.2.7. Determination of Drug Release Kinetics and Mathematical Modeling

Drug release from the nano-emulgel formulations was assessed using the cellulose dialysis membrane method. Dialysis membranes (molecular weight cutoff: 12,000 Da) were pre-hydrated in simulated salivary fluid (SSF, pH 6.8 ± 0.05) for 24 h prior to use. For each test, 1 mL of the nano-emulgel was placed inside the pre-hydrated membrane, which was securely tied at both ends. The sealed membrane was then immersed in SSF and placed in an orbital shaker incubator maintained at 37 °C (LM-530-2, MRC Laboratory Instruments Ltd., Hahistadrut, Holon, Israel) and agitated at 50 rpm. At predetermined time intervals (0.5, 1, 2, 4, 6, 8, 10, and 12 h), 3 mL of the release medium was withdrawn and immediately replaced with fresh SSF to maintain sink conditions. The absorbance of the oxidized azithromycin was measured at 482 nm using a UV–Visible spectrophotometer (Varian Cary UV–Visible Spectrophotometer Con 50, Agilent Technologies, Santa Clara, CA, USA). Drug release studies were conducted in triplicate (n = 3) for all previously identified stable formulations. The release kinetics were analyzed using zero-order, first-order, and Korsmeyer–Peppas models to elucidate the mechanism of drug release [61].

#### 4.2.8. Evaluation of Antimicrobial Activity

The broth dilution method was used to determine the MIC values of the nano-emulgels against various oral pathogens, namely *S. mutans* (ATCC 25175)*, A. actinomycetemcomitans* (ATCC 33384), *L. acidophilus* (ATCC 4356), and *L. casei* (ATCC 25598), all obtained from LGC Standards, South Africa. *S. mutans*, *L. acidophilus*, and *L. casei* were grown in Mueller Hinton Broth with 5% yeast extract supplemented with Hemophilus supplement, whereas *A. actinomycetemcomitans* was grown in Tryptone Soya broth. Each formulation started with an azithromycin concentration of 100 µg/mL within the nano-emulgel. This was added to 100 µL of appropriate media for the pathogen in a 96-well plate, resulting in a final azithromycin concentration of 0.2 µg/mL in the last row. Two-fold dilutions were then performed in the 48-well plate, followed by incubation at 37 °C with 5% CO_2_. *Streptococcus*, *L. acidophilus*, and *L. casei* were all incubated for 24 h at 37 °C, 5% CO_2_, and *A. actinomycetemcomitans* was incubated at the same environmental conditions as the other strains but for 2–5 days. After incubation, 40 µL of p-iodnitrotetrazolium salt was added to all wells as an indicator to detect viable microbial growth and to determine the MICs through visual inspection [62]. This study was performed in triplicate on consecutive days.

#### 4.2.9. Assessment of Rheological Properties

The rheological behavior of the nano-emulgel and its corresponding foam was evaluated using a Haake Modular Advanced Rheometer System II (Thermo Fisher Scientific, Johannesburg, South Africa). Measurements were conducted using a cone-plate geometry (Rotor C35/1, D = mm, 1 Titan) with a fixed gap of 0.050 mm. For testing, 1 mL of the nano-emulgel and two actuated pumps of the foam (equivalent to approximately 2–2.5 mL of liquid) were placed on the rheometer stage. Samples were subjected to a controlled shear rate ranging from 0.1 to 50 s^−1^ over a duration of 500 s to generate hysteresis loops and assess thixotropic behavior.

#### 4.2.10. Determination of Foam Expansion and Stability

Foam expansion refers to the ratio of foam volume after foam generation to the volume of liquid used to generate the foam [63]. Foam expansion and foam stability were determined by actuating each formulation using a foam actuator into a 100 mL beaker, which was then immediately sealed to prevent evaporation and external interference. The actuator was pumped five times (to actuate 5 mL of nano-emulgel into foam) for each formulation to ensure consistency, and the initial volume was noted. The collapse of the foam was observed for 15 min, after which the final foam volume was recorded. Foam expansion, stability, and stability index were calculated using the following formulas:

$$Foam\;expansion\left(\%\right)=\;\frac{volume\;of\;foam-volume\;of\;liquid\;actuated\;}{volume\;of\;liquid\;actuated}\times \;100$$

$$Foam\;stability\;\left(\%\right)\;=\;\frac{volume\;of\;final\;volme}{volume\;of\;initial\;foam}\times 100$$

$$Stability\;index=\frac{initial\;particle\;size-change\;in\;particle\;size}{initial\;particle\;size}\times 100$$

#### 4.2.11. Assessment of Foam Textural Properties

The texture properties of the foam actuated from the nano-emulgels were evaluated using a TA.XT Plus Texture Analyzer (Stable Micro Systems, Surrey, UK) equipped with a 100 mm flat cylindrical probe. The probe was used to compress the foam samples to a strain of 80%. The test parameters included a pre-test speed of 0.5 mm/s, a test speed of 1.0 mm/s, and a post-test speed of 0.5 mm/s. Spreadability and adhesiveness were determined from the force–time graph, where the positive peak indicated spreadability and the negative peak indicated adhesiveness. For each measurement, a single pump of foam was used, and all tests were performed in triplicate (n = 3).

#### 4.2.12. Determination of Ex Vivo Drug Permeation

Porcine buccal mucosa was cleaned and cut into 3 cm × 2 cm pieces. The study was conducted using the Franz Diffusion Cell (FDC) apparatus (PermeGear Inc., Bethlehem, PA, USA), with the receptor chamber containing phosphate buffer saline (PBS) of pH 6.8 ± 0.05. To simulate the oral environment, the donor chamber contained 1 mL of simulated salivary fluid with the nano-emulgel-actuated foam (equivalent to 1 mL of nano-emulgel formulation). The buccal mucosa was placed between the donor and receptor chambers, and the permeability test was carried out over 6 h. Samples were then analyzed using UV-vis at 482 nm (Varian Cary UV–Visible Spectrophotometer Con 50, Agilent Technologies, Santa Clara, CA, USA) to detect the quantity of azithromycin permeation.

### 4.3. Statistical Analysis

All data were expressed as mean values ± standard deviation. Statistical analysis was performed using the Student’s t-test, and a statistical significance of *p* < 0.05.

## Figures and Tables

**Table 1:** Description of vibrations of various functional groups in azithromycin versus the blank and loaded foams with mannitol and xanthan gum.

Description of Vibrations	Wavenumber (cm^−1^)		
	Pure Azithromycin	Blank Formulation	Loaded Formulation
O-H stretching of the sugar group	3491	3313	3363
C-H stretching of the alkyl group	2974	Absent	2924
C=O stretching of the carbonyl group from the lactone ring and carboxylic acid groups in the fatty chains of the nano-emulgels	1719	Absent	1715
C-O-C ether stretching	1189	Absent	1183
N-H stretching	3249	Absent	Absent

**Table 2:** Comparison of the size, polydispersity (PDI), and zeta potential of the nano-emulsions and nano-emulgels before and after stability tests.

Formulation	Before Stability Testing				After Stability Testing				
	Appearance	Size (d.nm)	PDI	Zeta Potential	Appearance	Size	PDI	Zeta Potential	Stability Index (%)
Blank nano-emulsions									
F3:7	Clear	15.10 ± 0.03	0.22 ± 0.38	−3.34 ± 1.60	Clear	19.26 ± 0.70	0.24 ± 0.09	−0.15 ± 2.10	78.40
F4:6	Clear	11.60 ± 1.20	0.04 ± 0.05	−5.53 ± 1.45	Clear	11.53 ± 0.56	0.21 ± 0.12	0.61 ± 1.50	99.40
F5:5	Clear	18.25 ± 0.26	0.32 ± 1.36	−6.59 ± 0.98	Clear	11.24 ± 0.15	0.16 ± 0.16	0.43 ± 0.50	61.59
F6:4	Clear	16.25 ± 0.55	0.22 ± 0.51	−8.34 ± 0.35	Clear	10.07 ± 0.31	0.12 ± 0.45	−0.54 ± 0.66	61.97
F7:3	Clear	11.36 ± 0.37	0.11 ± 0.48	−8.5 ± 0.51	Clear	12.07 ± 0.80	0.24 ± 0.90	−0.46 ± 0.41	93.26
F8:2	Clear	18.21 ± 1.11	0.45 ± 0.16	−10.7 ± 1.05	Clear	14.18 ± 0.41	0.23 ± 0.51	−0.29 ± 1.03	77.87
F9:1	Clear	14.42 ± 0.21	0.36 ± 0.66	−7.1 ± 1.30	Clear	12.71 ± 1.00	0.26 ± 0.33	−1.10 ± 0.80	84.40
**Loaded nano-emulsions**									
F5:5	Clear	11.08 ± 0.31	0.123 ± 0.12	−5.32 ± 0.51	Clear	11.19 ± 0.61	0.159 ± 0.21	−7.44 ± 1.20	99.00
F6:4	Clear	11.24 ± 0.65	0.092 ± 0.21	−6.58 ± 0.59	Clear	11.37 ± 0.97	0.121 ± 0.09	−9.29 ± 1.05	98.90
F7:3	Clear	10.65 ± 0.88	0.087 ± 0.25	−8.61 ± 0.34	Clear	12.07 ± 0.54	0.238 ± 0.80	−11.3 ± 0.50	88.20
F8:2	Clear	10.78 ± 0.16	0.077 ± 0.38	−8.06 ± 0.22	Clear	12.13 ± 0.26	0.230 ± 0.51	−12.0 ± 0.92	88.90
F9:1	Clear	10.66 ± 0.18	0.055 ± 0.10	−7.89 ± 0.40	Clear	17.07 ± 0.61	0.260 ± 0.12	−13.0 ± 0.56	62.40
**Blank nano-emulgels with xanthan gum and mannitol**									
F5:5	Clear	12.36 ± 0.25	0.227 ± 0.20	−7.62 ± 1.2	Clear	12.23 ± 0.88	0.215 ± 0.30	−8.65 ± 1.51	98.90
F6:4	Clear	17.10 ± 0.33	0.249 ± 0.34	−6.52 ± 1.65	Clear	11.55 ± 0.51	0.092 ± 0.37	−9.38 ± 1.02	67.50
F7:3	Clear	11.18 ± 0.65	0.114 ± 0.12	−8.07 ± 1.21	Clear	11.64 ± 0.63	0.145 ± 0.66	−22.20 ± 0.50	96.00
F8:2	Clear	17.88 ± 0.13	0.229 ± 0.40	−9.43 ± 1.11	Clear	12.24 ± 0.40	0.16 ± 0.27	−23.10 ± 1.41	68.50
F9:1	Clear	11.91 ± 0.15	0.162 ± 0.38	−8.14 ± 0.80	Clear	11.86 ± 0.72	0.132 ± 0.33	−33.00 ± 0.87	99.60
**Loaded nano-emulgels with xanthan gum and mannitol**									
F5:5	Clear	11.33 ± 0.22	0.099 ± 0.05	−13.50 ± 0.99	Clear	13.01 ± 0.16	0.234 ± 0.55	−11.70 ± 1.18	87.10
F6:4	Clear	17.92 ± 0.58	0.230 ± 0.13	−23.40 ± 0.84	Clear	12.15 ± 0.25	0.143 ± 0.29	−10.10 ± 0.96	67.80
F7:3	Clear	16.37 ± 0.31	0.272 ± 0.06	−18.40 ± 0.24	Clear	13.58 ± 0.41	0.232 ± 0.32	−22.90 ± 0.74	83.00
F8:2	Clear	14.78 ± 0.69	0.262 ± 0.19	−6.94 ± 1.33	Clear	13.07 ± 0.07	0.231 ± 0.17	−13.70 ± 1.70	88.40
F9:1	Clear	10.66 ± 0.41	0.055 ± 0.24	−7.89 ± 1.15	Clear	23.50 ± 0.65	0.718 ± 0.89	−14.10 ± 0.95	45.40

**Table 3:** A comparison of the nano-emulsions and nano-emulgels before and after each stability test.

Formulations	Initial Appearance	Centrifugation	Heat–Cool Cycle	Freeze–Thaw Cycle	Initial Appearance	Centrifugation	Heat–Cool Cycle	Freeze–Thaw Cycle
	Blank nano-emulsions					Loaded nano-emulsions		
F 1:9	X	-	-	-	X	-	-	-
F 2:8	X		-	-	X	-	-	-
F 3:7	✓	✓	✓	✓	X	-	-	-
F 4:6	✓	✓	✓	✓	X	-	-	-
F 5:5	✓	✓	✓	✓	✓	✓	✓	✓
F 6:4	✓	✓	✓	✓	✓	✓	✓	✓
F 7:3	✓	✓	✓	✓	✓	✓	✓	✓
F 8:2	✓	✓	✓	✓	✓	✓	✓	✓
F 9:1	✓	✓	✓	✓	✓	✓	✓	✓
	**Blank nano-emulgels with xanthan gum and mannitol**				**Loaded nano-emulgels with xanthan gum and mannitol**			
F 1:9	X	-	-	-	X	-	-	-
F 2:8	X	-	-	-	X	-	-	-
F 3:7	X	-	-	-	X	-	-	-
F 4:6	X	-	-	-	X	-	-	-
F 5:5	✓	✓	✓	✓	✓	✓	✓	✓
F 6:4	✓	✓	✓	✓	✓	✓	✓	✓ *
F 7:3	✓	✓	✓	✓	✓	✓	✓	✓
F 8:2	✓	✓	✓	✓	✓	✓	✓	✓
F 9:1	✓	✓	✓	✓	✓	✓	✓	✓

* Indicates that the nano-emulgel showed a slight haziness after completing the freeze–thaw cycle. However, since the stability index remained within acceptable limits, the formulation was considered stable and deemed suitable for further studies.

**Table 4:** Drug release kinetic modeling data of azithromycin release from the nano-emulgels.

	Zero-Order Kinetics		First-Order Kinetics		Korsmeyer–Peppas			Best Fit Model
Formulation	r^2^	K_0_ (h^−1^)	r^2^	K_1_ (h^−1^)	r^2^	K_KP_ (h^−n^)	n	
F 5:5	0.95	0.05	0.91	1.62	0.97	0.57	1.44	Korsmeyer–Peppas
F 6:4	0.98	0.09	0.66	2.79	0.92	0.46	0.95	Zero-order kinetics
F 7:3	0.72	0.06	0.51	2.92	0.96	0.51	0.35	Korsmeyer–Peppas
F 8:2	0.91	0.03	0.99	2.19	0.97	0.80	0.86	Korsmeyer–Peppas
F 9:1	0.90	0.08	0.98	6.00	0.88	0.12	0.43	First-order kinetics

**Table 5:** Minimum inhibitory concentrations of nano-emulgels’ antimicrobial activity against oral pathogens.

Samples (µg/mL)	*S. mutans* (ATCC 25175)	*L. acidophilus* (ATCC 4356)	*L. casei* (ATCC 25598)	*A. actinomycetemcomitans* (ATCC 33384)
**5:5 blank**	1.56 ± 0	1.56 ± 0	1.56 ± 0	1.56 ± 0
**5:5 loaded**	0.39 ± 0	1.56 ± 0	0.78 ± 0	0.59 ± 0.276
**6:4 blank**	1.56 ± 0	1.56 ± 0	1.56 ± 0	1.56 ± 0
**6:4 loaded**	0.78 ± 0	0.78 ± 0	3.13 ± 0	0.78 ± 0
**7:3 blank**	1.56 ± 0	1.56 ± 0	1.56 ± 0	1.56 ± 0
**7:3 loaded**	6.25 ± 0	1.56 ± 0	6.25 ± 0	1.17 ± 0.552
**8:2 blank**	1.56 ± 0	1.56 ± 0	1.56 ± 0	1.56 ± 0
**8:2 loaded**	25.00 ± 0	1.56 ± 0	12.50 ± 0	2.35 ± 1.110
**9:1 blank**	0.78 ± 0	0.78 ± 0	0.78 ± 0	0.78 ± 0
**9:1 loaded**	25.00 ± 0	1.56 ± 0	25.00 ± 0	3.13 ± 0
**Culture control**	>250	>250	>250	>250
**Positive control (Azithromycin at starting conc. 0.1 µg/mL)**	3.91 ± 1.13	6.25 ± 0	4.69 ± 2.206	4.69 ± 2.206
**Negative control**	>250	>250	>250	>250

Grey shaded rows indicate azithromycin-loaded nano-emulgels.

**Table 6:** Composition of the various nano-emulsion formulations.

Formulation	Olive Oil (% *v*/*v*)	Tween 80 (% *v*/*v*)	Labrasol (% *v*/*v*)	Lecithin (% *w*/*v*)	Deionized Water (% *v*/*v*)
F1:9	4.55	4.55	40.91	4.55	45.44
F2:8	5.00	10.00	40.00	5.00	40.00
F3:7	4.30	13.00	30.40	4.30	48.00
F4:6	3.33	13.33	20.00	3.33	60.10
F5:5	2.38	11.9	11.9	2.38	71.44
F6:4	2.38	14.28	9.53	2.38	71.43
F7:3	1.92	13.46	5.77	1.92	76.93
F8:2	1.92	15.38	3.85	1.91	76.93
F9:1	1.92	17.31	1.92	1.92	76.93

## Data Availability

The raw data presented in this study are not publicly available but may be available for researchers on request from the corresponding author after a special review that includes approval of the research project.

## Supplementary Materials

The following supporting information can be downloaded at https://www.mdpi.com/article/10.3390/gels11050373/s1, Figure S1: Solubilization ability of azithromycin in potential oils, surfactants, and co-surfactants; Figure S2: Chemical schematics; Figure S3: FTIR spectra of nano-emulgel components; Figure S4: FTIR spectra of blank and azithromycin-loaded foams with mannitol and xanthan gums; Figure S5: DLS plots; Figure S6: Digital photograph of foam behavior after texture analysis test; Figure S7: Microscopic photograph of nano-emulgel actuated foam; Figure S8: Drug release kinetics mathematical modeling plots.

## Abbreviations

The following abbreviations are used in this manuscript:

AUC	Area under the curve
AZM	Azithromycin
BoP	Bleeding on probing
CAL	Clinical attachment loss
CFU	Colony-forming units
EE	Encapsulation efficiency
FDA	Food and Drug Administration
FDC	Franz diffusion cell
FTIR	Fourier transform infrared spectroscopy
GIT	Gastrointestinal tract
HIV	Human immunodeficiency virus
IL	Interleukin
MICs	Minimum inhibitory concentrations
PDI	Poly dispersity index
Smix	Surfactant mixture
SSF	Simulated salivary fluid
