# Supplementary material for: Design of a Foam-Actuated Nano-Emulgel for Perioceutic Drug Delivery: Formulation, Characterization, and Antimicrobial Efficacy
Source: Gels. 2025 May 20;11(5):373. doi: 10.3390/gels11050373 (PMC12111423; doi:10.3390/gels11050373)
Supplement: Supplementary file 1 [file gels-11-00373-s001.zip › gels-3610896-supplementary.pdf]

Supplementary Materials

Design of a Foam-Actuated Nano-Emulgel for Peri-oceutic Drug Delivery: Formulation, Characterization, and Antimicrobial Efficacy

Theresa P. K. Varughese [1], Poornima Ramburrun [1], Nnamdi I. Okafor [1], Sandy van Vuuren [2] and Yahya E. Choonara [1,3,*]

[1] Wits Advanced Drug Delivery Platform Research Unit, Department of Pharmacy and Pharmacology,
School of Therapeutic Sciences, Faculty of Health Sciences, University of the Witwatersrand, 7 York Road,
Parktown, Johannesburg 2193, South Africa; poornima.ramburrun@wits.ac.za (P.R.); nnamdi.okafor@wits.ac.za (N.I.O.)

[2] Department of Pharmacy and Pharmacology, School of Therapeutic Sciences, Faculty of Health Sciences, University of the Witwatersrand, 7 York Road, Parktown, Johannesburg 2193, South Africa;
sandy.vanvuuren@wits.ac.za

[3] Wits Infectious Diseases and Oncology Research Institute, Faculty of Health Sciences, University of the Witwatersrand, 7 York Road, Parktown, Johannesburg 2193, South Africa

* Correspondence: yahya.choonara@wits.ac.za; Tel.: +27-11-717-2052

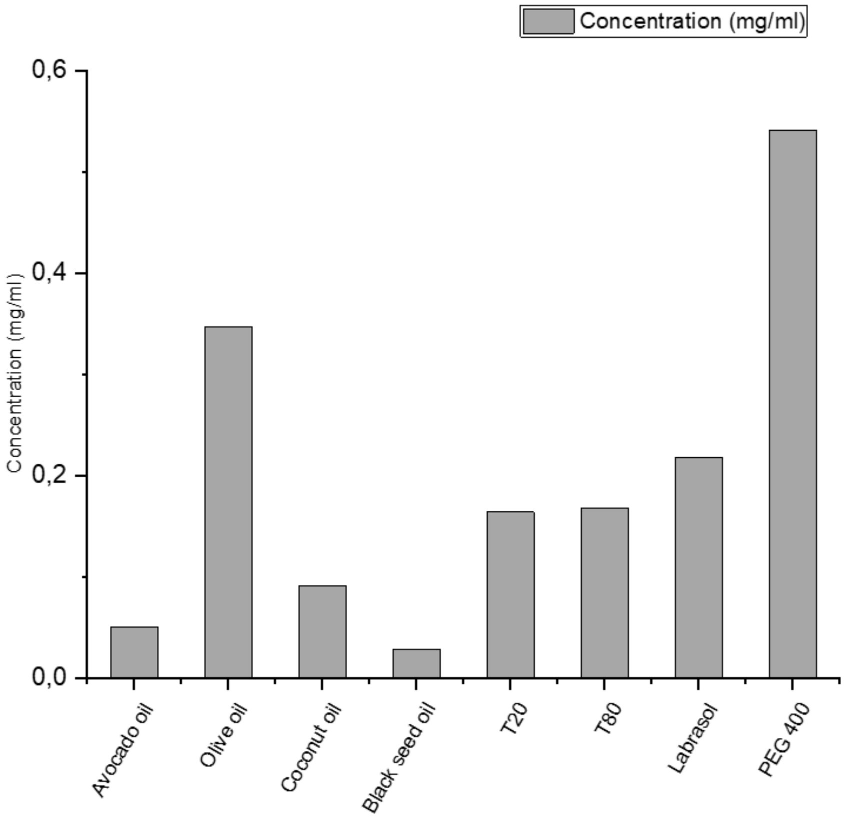

Figure S1. Solubilization ability of azithromycin in potential oils, surfactants and co-surfactants.

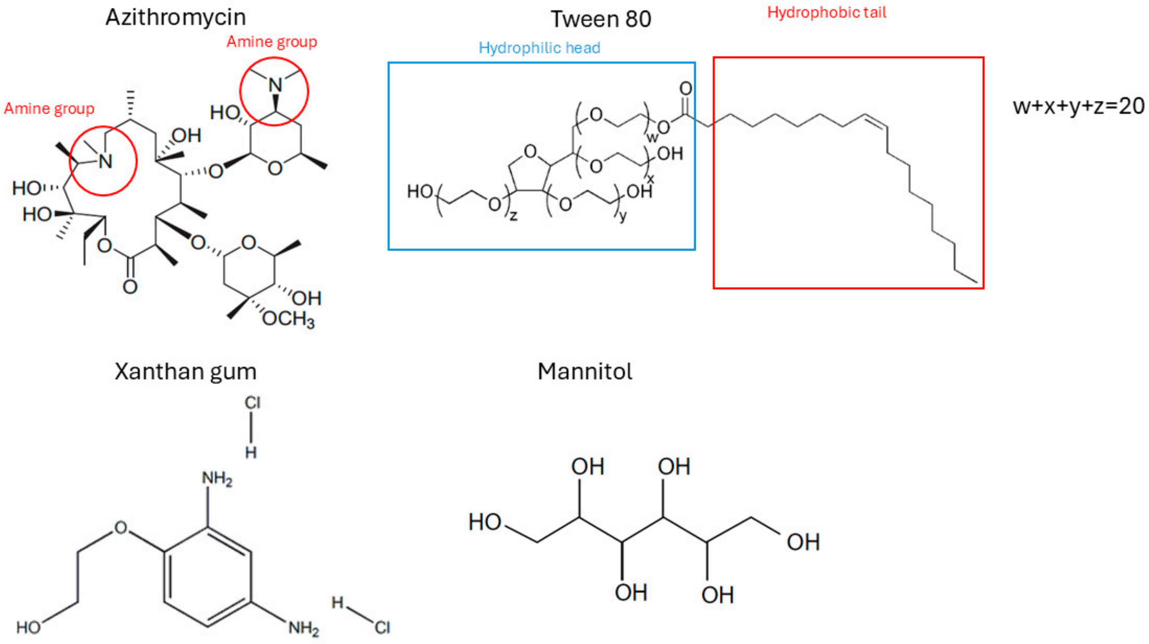

Figure S2: Chemical structures of Azithromycin and the excipients used in nano-emulgel formation.

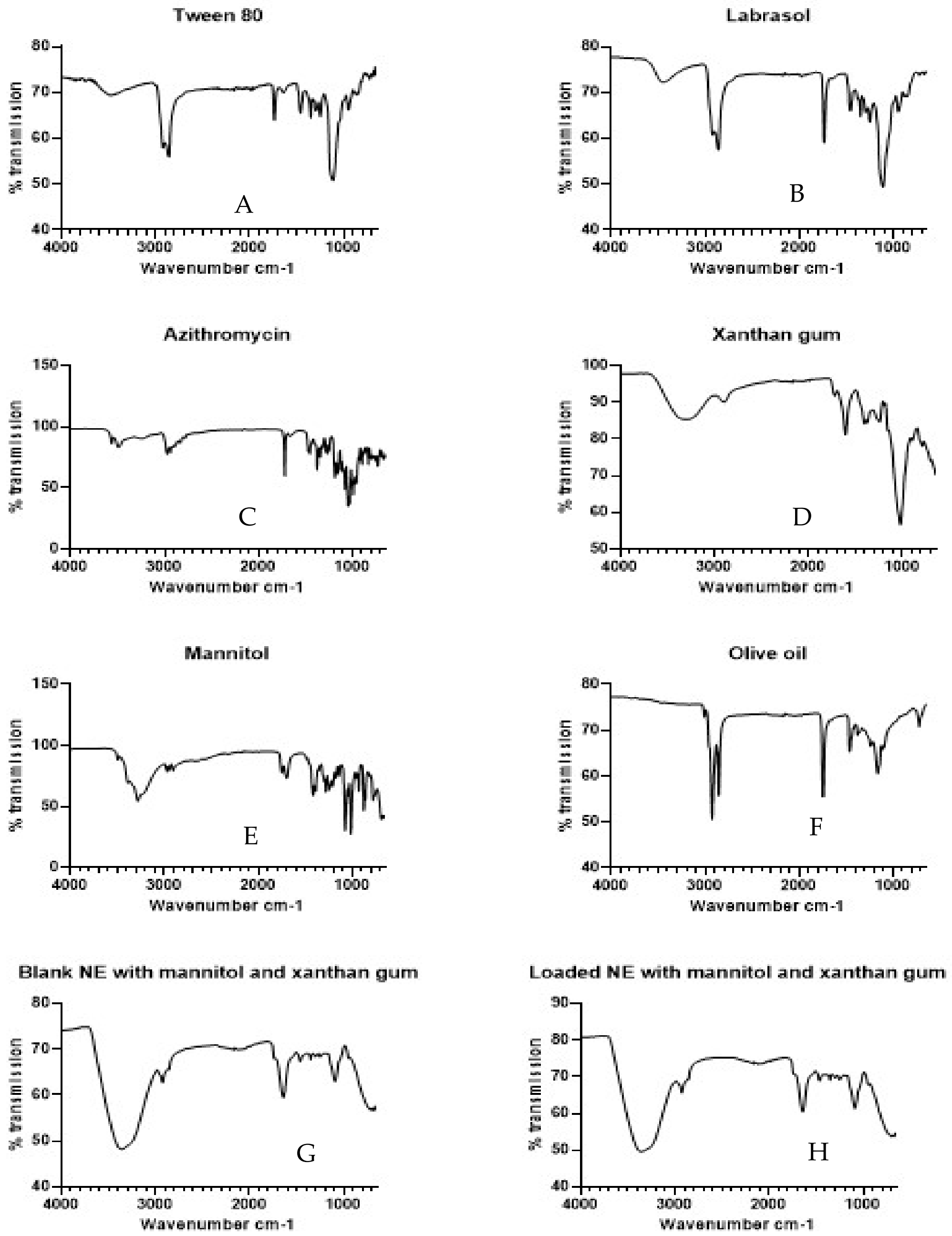

Figure S3. FTIR spectra of nano-emulgel components: A) Tween 80, B) Labrasol, C) Azithromycin, D) Xanthan gum, E) Mannitol, F) Olive oil, G) Blank nano-emulgel with mannitol and xanthan gum and lastly H) Loaded nano-emulgel with mannitol and xanthan gum

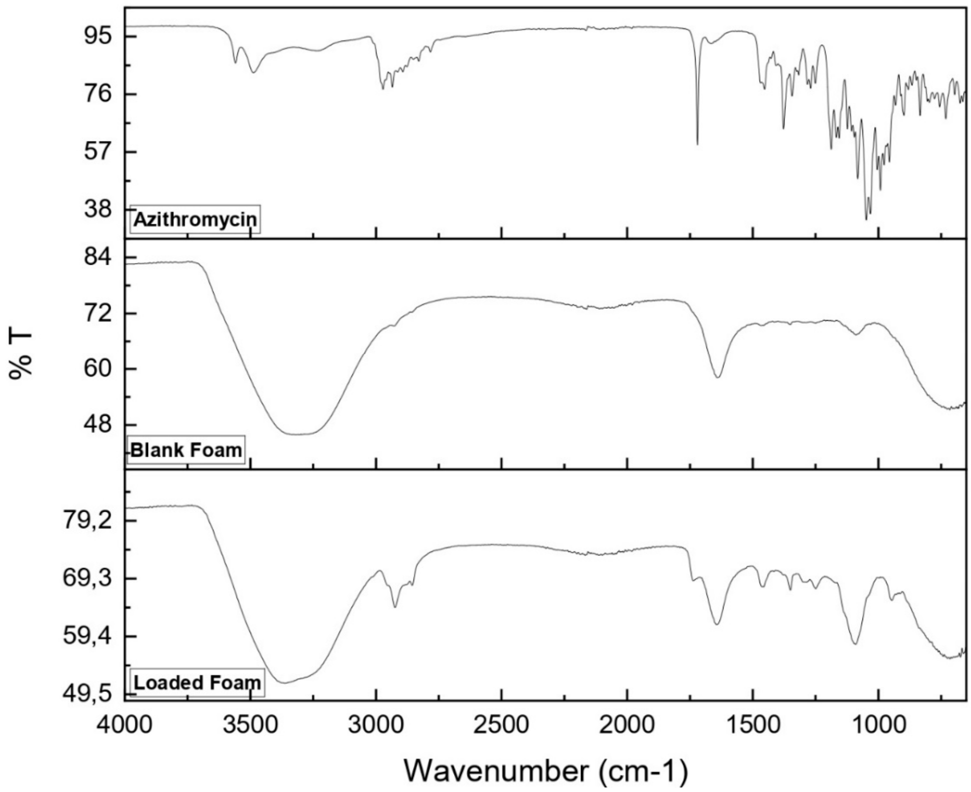

Figure S4. FTIR spectra of blank and azithromycin-loaded foams with mannitol and xanthan gums.

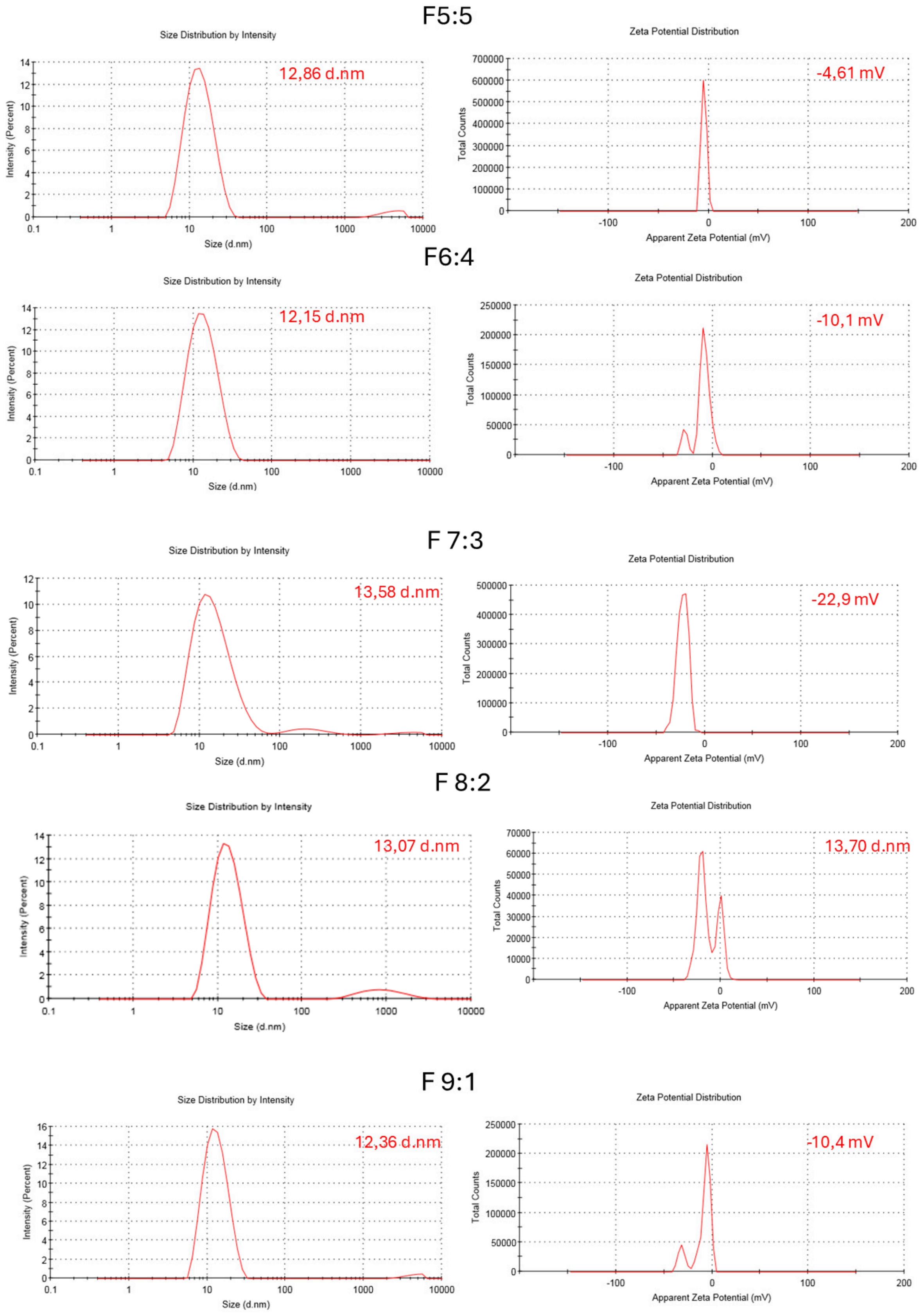

Figure S5: Dynamic Light Scattering(DLS) curves depicting average particle size and zeta-potential

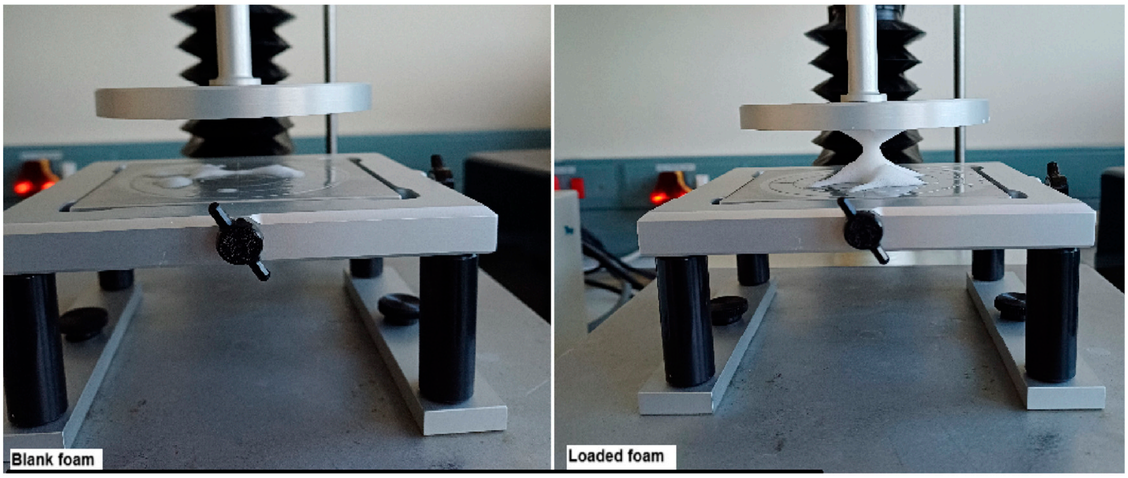

Figure S6. Digital photograph of foam behavior after texture analysis test.

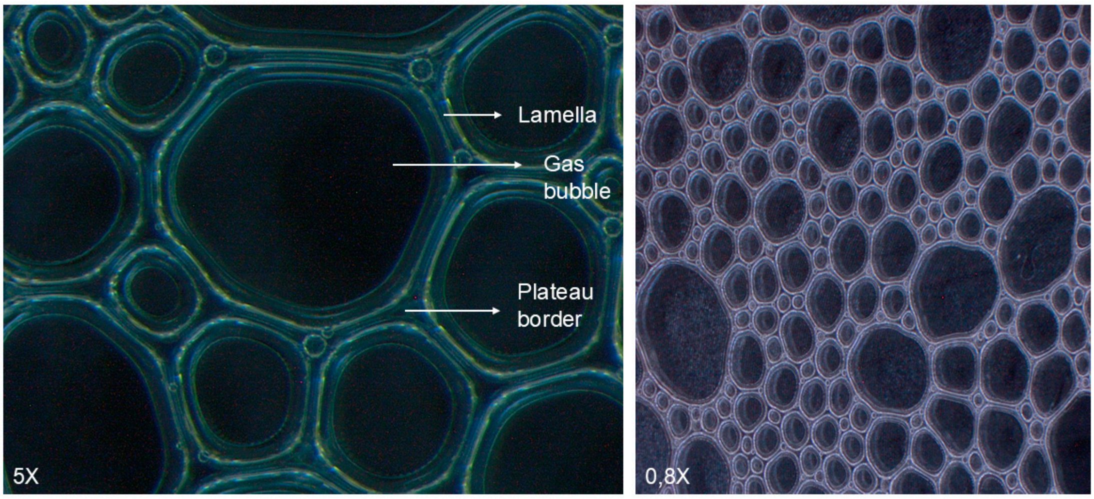

Figure S7. Microscopic photograph of nano-emulgel actuated foam.

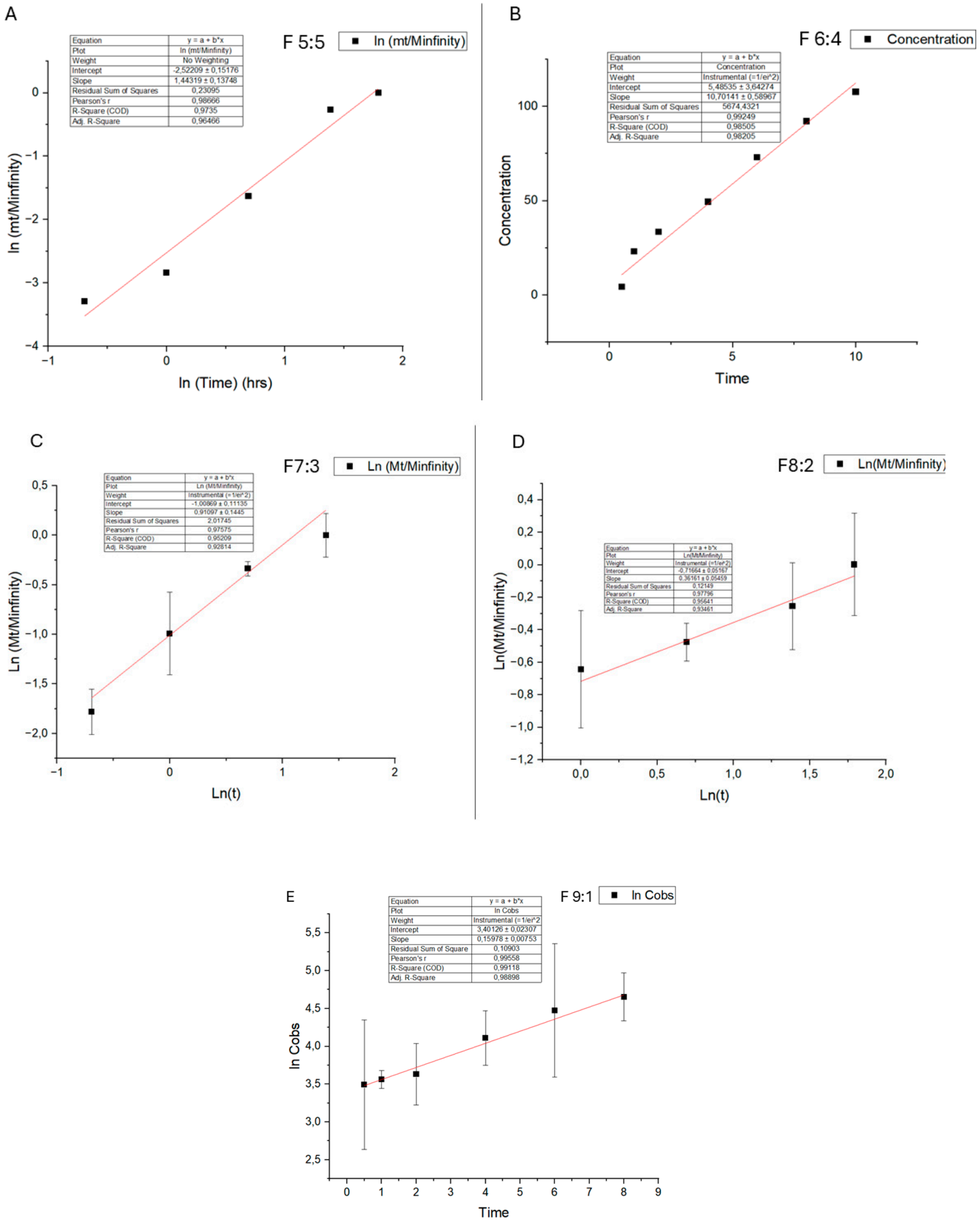

Figure S8: A) Korsmeyer-peppas release kinetics of F 5:5, B) Zero-order release kinetics of F6:4, C and D) Korsmeyer-Peppas release kinetics for F 7:3 and F 8:2 respectively, and lastly E) depicting first order kinetics of F 9:1.
